# Factors that influence the uptake of precision-guided treatment recommendations in paediatric cancer: a systematic review

**DOI:** 10.1038/s41416-025-03171-6

**Published:** 2025-09-24

**Authors:** Xian Zou, Tarini Srivastava, Kate Hetherington, Alice Yu, Glenn M. Marshall, Marion K. Mateos

**Affiliations:** 1 Behavioural Sciences Unit, Kids Cancer Centre, Sydney Children’s Hospital, Sydney, NSW Australia; 2 School of Clinical Medicine, UNSW Medicine and Health, Discipline of Paediatrics, UNSW Sydney, Sydney, NSW Australia; 3 Kids Cancer Centre, Sydney Children’s Hospital Randwick, Sydney, NSW Australia; 4 Centre for Health Economics Research and Evaluation (CHERE), University of Technology Sydney, Sydney, NSW Australia; 5 Children’s Cancer Institute, Lowy Cancer Research Centre, UNSW, Sydney, NSW Australia

**Keywords:** Targeted therapies, Paediatric cancer

## Abstract

**Abstract:**

Despite potential benefits, the clinical uptake of precision-guided treatment (PGT) in paediatric oncology remains low. This systematic review aimed to identify factors affecting the adoption of PGT recommendations for children with cancer. Five databases (EMBASE, CINAHL, PubMed, PsycINFO, Scopus) were systematically searched. Qualitative and quantitative studies involving patients aged 0–21 years were included. Two reviewers conducted screening and data extraction. Seventeen studies, all from developed countries, met the criteria: 10 prospective, 4 retrospective, and 3 cross-sectional, involving 3976 patients, 227 healthcare professionals, 189 parents, and 532 community members. Most studies were quantitative, collating factors from clinical records; two qualitative studies used interviews to explore clinician and parent perspectives. Three key domains of factors were identified: (i) decision maker-related characteristics; (ii) decision-specific criteria; and (iii) contextual factors. Common factors included limited drug/clinical trial access, ongoing alternative treatments, high costs, and patient or family preferences. These interlinked factors may affect uptake individually and collectively. To our knowledge, this is the first review to map factors influencing the implementation of PGT in paediatric cancer. Further research is needed to clarify the relative importance of these factors and to better understand oncologists’ perspectives when making recommendations in partnership with patients and families.

**Registration:**

PROSPERO database (CRD42023410199)

## Introduction

Globally, 300,000 children aged 0–19 years are diagnosed with cancer annually [1]. Between 2020 and 2050, 13.7 million new cases are expected, but 45% will go undiagnosed due to health system limitations, especially in low-income countries [2]. In high-income countries, cancer is the leading cause of death in children, highlighting the need for improved diagnostic and treatment strategies [3]. The use of genomic testing has increased in both adult and paediatric oncology, enabling mutation identification, personalised therapies, and improved outcomes, which is crucial for paediatric cancer with limited treatment options [4].

Paediatric precision oncology is a rapidly evolving field that aims to tailor cancer treatment to an individual patient’s unique genetic and molecular profile. In this review, precision medicine is defined as the process of using sequencing information or high throughput drug screening to identify treatment recommendations. This personalised approach promises significant improvements in treatment efficacy and potential reductions in conventional treatment side effects. It offers hope for children diagnosed with cancer [5], particularly poor prognosis cancers. However, despite precision medicine offering more accurate diagnosis, prognostication and potential treatment benefits to patients, the actual clinical uptake rate of precision-guided treatment (PGT) within clinical trials remains low around the world [6–8].

To facilitate the implementation of PGT in paediatric oncology, it is essential to understand the decision-making process regarding the adoption of these treatments. There is no optimal uptake rate for PGT, as the understanding of cancer genetics continues to evolve, along with the evidence base and the availability of therapeutic agents. Considering this, it is essential to prioritise understanding the decision-making processes and providing appropriate support for both families and healthcare professionals as they navigate these complex decisions. Prior research on implementing new drugs or treatments in cancer clinical practice (not specific to paediatrics) has identified a number of factors including, but not limited to, clinical and genetic considerations, the expertise and perspectives of healthcare providers, parental and familial influences, emotions, patient-doctor relationship, ethical and socioeconomic aspects, drug accessibility; and the broader health system and institutional environment [8–10].

The aim of this systematic review was to identify factors that influence decision-making regarding uptake of PGT recommendations in childhood cancer. We were specifically interested in understanding the influencing factors from 1. the treating clinician’s perspective, and 2. the patient/family perspective.

## Methods

### Protocol and registration

We followed the Preferred Reporting Items for Systematic Review and Meta-Analysis (PRISMA) guideline [11]. A protocol for this review was registered on PROSPERO (Registration number: CRD42023410199).

### Database search procedure

Two authors (XZ and MKM) developed the search strategy based on key words and key articles in the field. The search was conducted in five electronic databases: EMBASE via Ovid, CINAHL via EBSCO, PubMed, PsycINFO via Ovid, and Scopus. Hand-searching was conducted using Google Scholar, connected papers, reference lists, citations of included studies and relevant systematic reviews to identify relevant studies that were eligible.

The search strategy was developed in collaboration with a librarian at the University of New South Wales, Australia. The search was completed on 12^th^ January 2023. The search terms were developed across four categories: ‘precision medicine’, ‘children’, ‘cancer’ and ‘treatment decision making’, as summarised in Table 1.

**Table 1:** Search terms used in each database.

Treatment decision making	clinical decision making OR decision making OR choice behavio*r OR shared decision making OR patient preference OR stakeholder participation OR stakeholder engagement OR decision making
AND cancer	cancer OR Neoplasms OR oncology OR malignancy OR tumour
AND precision medicine	precision medicine OR personalized medicine OR molecular targeted therapy OR genomic medicine OR genetic testing OR whole genome sequencing OR genetics OR molecular profiling OR germline testing OR preclinical testing OR targeted therapy OR recommended treatment
AND children	infant OR child OR adolescent OR parents OR p*ediatrics OR p*ediatricians OR young adults OR AYA

### Eligibility criteria

Studies were included if the following criteria were met:

1. Original primary peer-reviewed research, including studies conducted in research or trial, clinical or hybrid contexts.
2. Obtained evidence about factors which impacted on medical specialists’/health professionals’ decisions to offer treatment recommendations, including parents’/patients’ contribution to this decision making, in the context of precision medicine.
3. Research articles that discuss treatment or change in treatment (e.g. maintain the current therapy/take up recommended treatment/stop therapy/ palliative approach).
4. Studies published in English.

Studies were excluded if they:

1. Were a systematic review or review article.
2. Did not have full text available.
3. Focused on populations where genetic testing was conducted for prenatal or reproductive decisions.

### Screening and data extraction

All articles were returned by the preliminary search through the 5 databases, and hand search through Google Scholar. The reference list of all included studies was searched for additional relevant paediatric precision medicine studies.

Following the removal of duplicates using the Rayyan programme (https://www.rayyan.ai/), two reviewers (XZ and TS) independently screened all the titles and abstracts.

Full text screening was conducted by two reviewers (XZ and TS) using Covidence. Any disagreements and conflicts were discussed first between the two primary reviewer, and if no consensus was reached then a third reviewer (MKM) was consulted before a final decision was made.

Types of data extracted involved cancer type, molecular analyses performed, turnaround time (TAT), tiering system used, whether a Molecular/Multidisciplinary Tumour Board (MTB) was used to provide PGT recommendations, and factors that influenced clinical uptake. Among these data, TAT refers to the duration from the patient sample collection to the generation of testing results by the laboratory [12]. A tiering system was used to rank the supporting evidence for PGT from the highest to the lowest, with an example from the Boston tiering system as follows: Tier 1, clinical evidence in the same cancer; Tier 2, clinical evidence in a different cancer; Tier 3, preclinical evidence in the same cancer; Tier 4, preclinical evidence in a different cancer type; and Tier 5, consensus opinion [6]. This hierarchy of evidence underscores the varying degrees of confidence in treatment recommendations, further complicating decision-making processes. The MTB is tasked with choosing tailored and focused individual treatment plans based on the molecular results.

### Quality assessment

Eligible studies were assessed for quality and risk of bias against the Mixed Methods Appraisal Tool (MMAT) [13]. The MMAT can be used to appraise both qualitative and quantitative articles, evaluating aspects including the research approach, methods, outcome measurements, statistical analysis, representativeness, risk of confounding, and nonresponse bias. Two authors (XZ) and (TS) assessed the 17 articles. Conflicts with scoring were addressed and settled via discussion, including with a third author (MKM) if required. The appraisal results are presented in Supplementary Table 1.

## Results

**Fig. 1:**
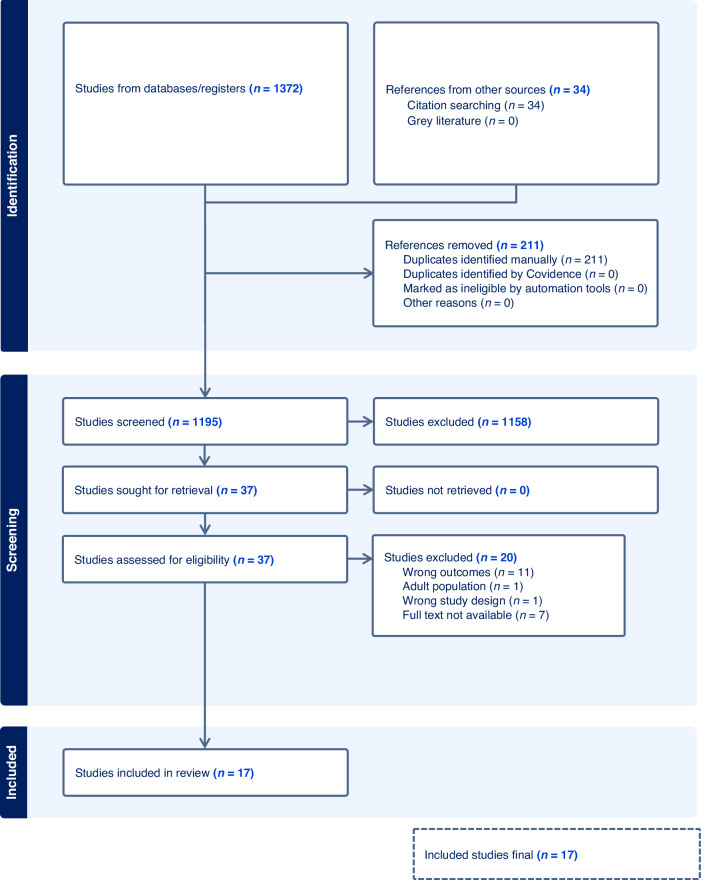
PRISMA flow diagram: search and selection process.

From 1372 articles returned by the search strategy, 1158 articles were excluded after title and abstract screening, and 20 articles were excluded after full text screening. This led to 17 eligible articles for which data were extracted. The study selection process is illustrated in the PRISMA flow diagram (Fig. 1). All 17 studies were of adequate-high quality based on the MMAT (Supplementary Table 1).

A meta-analysis was not feasible due to the heterogeneity of study design and methods used. Thus, a narrative synthesis was conducted to summarise the findings of the included studies, as shown in Table 2.

**Table 2:** Study characteristics.

First Author, Year	Country	Total No. of patients	Type of genomic testing	Cohort	Study type	Decision- making context (TAT)	Parents (n)	Tumour type	Age of children^#^	PGT from MTB	Clinicians /HCP (n)
Church et al. [7]	USA	345	RNA-seq; OncoPanel; RNA fusion panel	Primary high-risk; Relapse/Refractory	Prospective; Quantitative	Clinical record (Mean 42.4 d)	23	Extracranial solid tumours, sarcoma, Neuroblastoma	Median age 12 years, ≤ 27.5 years	Yes	N/A
Parsons et al. [23]	USA	1000	RNA-seq; DNA methylation profiling; IHC assays	Relapse/Refractory	Prospective; Quantitative	Clinical record ( ≤ 28 d)	23	Solid tumours, non- Hodgkin lymphomas or histiocytic disorders	1–21 years	No	N/A
Van Tilburg et al. [25]	Other^a^:	1051	lcWGS (Somatic); WES (Somatic); RNA- seq; DNA methylation profiling; RNA based gene expression array	Primary high-risk; Relapse/Refractory	Prospective; Quantitative	Clinical record ( ≤ 28 d)	312 (Pre), 157 (Post)	Rare tumours; Rare cancer	<21 years	Yes	N/A
Villani et al. [26]	Canada	300	Germline WGS; RNA-seq; pan- cancer DNA panel	Relapse/Refractory	Prospective; Quantitative	Clinical record (N/A)	570	Rare cancer, hard-to- treat cancer, metastatic, unresolved suspicion of cancer predisposition, CNS Tumours, Leukaemia, lymphoma, solid Tumours	Median age 7.1 years	Yes	N/A
Ortiz et al. [22]	USA	39	WES (Somatic); Targeted gene panel	Primary high-risk; Relapse/Refractory	Retrospective ; Quantitative	Clinical record (N/A)	16	Haematologic malignancies, solid tumours, brain tumours	Median age 13 years	Yes	N/A
Pincez et al. [24]	France/Europe	60	NGS DNA gene panel; aCGH	Primary high-risk; Relapse/Refractory	Prospective; Quantitative	Clinical record (Mean 42 + /- 16 d)	47	Expected survival rate <25%	Mean age 12 ± 5.7 years (range 0.1–21.5)	Yes	N/A
Mody et al. [20]	USA	102	WES (Somatic); RNA-seq	Relapse/Refractory	Prospective; Quantitative	Clinical record (Mean 54 d)	32	Rare cancer, With a suspected diagnosis of a neoplastic disorder	<25 years	Yes	N/A
Marks et al. [19]	USA	56	WES (Somatic); Germline WES; RNA-seq; DNA sequencing of 467 cancer associated genes	Primary high-risk; Relapse/Refractory; Other: Prognosis <50% overall survival at 5 years	Retrospective ; Quantitative	Clinical record ( ≤ 42 d)	121	High-risk haematologic malignancies and blood disorders; rare haematologic disorder or unusual presentation for age	Other^b^	Yes	N/A
Oberg et al. [21]	USA	101	WES (Somatic); Germline WES; RNA-seq; DNA methylation profiling	Primary high-risk; Relapse; Rare cancer	Retrospective ; Quantitative	Clinical record (Mean 60 d)	20	Cancer predisposition	Mean age 9.3 years; median age 8.0 years	Yes	N/A
George et al. [16]	UK	223	Germline WES; ctDNA; Paeds NGS	Relapse/Refractory	Prospective; Quantitative	Clinical record (N/A)	38	Solid tumours	<24 years	Yes	N/A
Harris et al. [27]	USA	100	Targeted NGS (OncoPanel)	Primary high-risk; Refractory	Prospective; Quantitative	Clinical record (N/A)	64	Extracranial solid tumours	<30 years	Yes	N/A
De Abreu Lourenco et al. [14]	Australia	N/A	Genomically guided approach (no specific information given)	Other^c^	DCE; Quantitative	Primary aim, interview, survey (N/A)	33	N/A	N/A	No	126
McCullough et al. [28]	USA	N/A	WES	Primary high-risk	Qualitative	Primary aim, semi-structured interviews (N/A)		CNS and non-CNS solid tumours	N/A	No	16
Harttrampf et al. [17]	France	75	WES (Somatic); RNA-seq	Incurable, Relapse/Refractory	Prospective; Quantitative	Clinical record ( ≤ 42 d)	19	Hard-to-treat cancer; CNS, Other: Solid malignancy	Median age 10.9 years (range 0.8–24.3)	Yes	N/A
Eaton et al. [15]	USA	33	Genomic profiling; Germline WGS; Other: Functional ex vivo drug sensitivity testing	Newly diagnosed; Relapse/Refractory	Retrospective ; Quantitative	Clinical record (N/A)	116	Haematologic malignancies, including ALL, AML, CML, MPAL, myelodysplasia, myelofibrosis, non- Hodgkin lymphoma and Hodgkin lymphoma	<25 years	Yes	N/A
McCarthy et al. [29]	Australia	1	Genome sequencing	Relapse/Refractory;	Qualitative	Primary aim, interview (N/A)	53	Hard-to-treat cancer	Range 1.8-19.3 years	No	22
Langenberg et al. [18]	Netherlands	253	lcWGS (Somatic); WES (Somatic); RNA- seq; Affymetrix gene expression; DNA methylation profiling	Primary high-risk; Relapse/Refractory	Prospective; Quantitative	Clinical record (Mean 42 d)	N/A	Extracranial solid tumour, CNS, Haematological malignancies	Median 13.1 years (range: 0.75 - 23)	Yes	44

Age of children#: Either median age or range of age of patients at diagnosis were provided according to the data from included studies.

‘Decision-making context’ (TAT)—the decision-making context refers to decision-making related data were collected from clinical record, interview or survey, including the turnaround time (TAT) of sequencing results, the time recorded from patient sample collection to testing report generated by the lab.

*RNA-seq* mRNA sequencing. Targeted DNA panel (OncoPanel), *lcWGS* low-coverage Whole Genome Sequencing, *WES* whole exome sequencing, *aCGH* array comparative genomic hybridisation, *ctDNA* circulating tumour DNA, *Paeds* paediatrics, *NGS* next generation sequencing, *DCE* discrete choice experiment, *CNS* central nervous system tumour, *ALL* acute lymphoblastic leukaemia, *AML* acute myeloid leukaemia, *CML* chronic myeloid leukaemia, *MPAL* mixed-phenotype acute leukaemia, *WGS* whole genome sequencing, *N/A* not applicable.

Other^a^: Patients were enroled in a total of 72 centres in the following eight countries: Austria (*n* = 5), Finland (*n* = 5), Germany (*n* = 396), Greece (*n* = 3), Poland (*n* = 2), Sweden (*n* = 36), Switzerland (*n* = 13), and the Netherlands (*n* = 59).

Other^b^: Mean (median). Lymphoid, 9.5 [8]; Myeloid, 9.9 [10]; Lymphoma, 13.8 [17]; Histiocytic disorder, 5.7 [2].

Other^c^: Participants are different stakeholders in the context of difficult-to-treat childhood cancer, including health care providers (HCPs), parents of childhood cancer survivors, and general community members.

### Study characteristics

All studies were conducted between 1 May 2012 and 1 April 2021. No conflicts of interest were reported among these studies.

All 17 eligible studies were conducted in developed countries, mostly in North America (9 in the USA, 1 in Canada). The remaining seven studies were led from Australia (2 studies), France (2 studies), Germany (1 multi-institutional study), Netherlands (1 study), and UK (1 study) (Table 2).

Methodologies were diverse with 15 quantitative studies [7, 14–27] and 2 qualitative studies [28, 29]. This included 13 feasibility studies, 1 cohort study and 1 Discrete Choice Experiment (DCE) survey. Among the 15 quantitative studies, 13 studies involved paediatric cancer patients and assessed the feasibility of molecular profiling and/or identifying actionable alterations and making PGT recommendations to paediatric patients with cancer. In these studies, information related to influencing factors regarding treatment decision-making was often reported briefly and lacked comprehensive detail. One cohort study reported that, with highest target priority level of recommendation, 47.6% (20 out of 42) patients took up PGT recommendation; the study also compared the clinical benefit (progression-free survival days) between patients who took up PGT recommendation and other patients [27]. The DCE survey aimed to quantify the importance of those influencing factors among healthcare professionals, parents and community members [14]. The remaining 2 qualitative studies interviewed paediatric healthcare professionals and parents to investigate the influencing factors in detail [28, 29].

### Sample characteristics

A total of 3976 patients, 227 clinicians, 189 parents or caregivers, and 532 general community participated in the included studies. Healthcare professionals included paediatric oncologists, physicians, and clinicians (Table 2). The patient populations in the included studies consisted of individuals with high-risk cancers or those with relapsed or refractory disease following conventional therapy, both associated with a limited likelihood of cure (Table 2).

Among patients for whom a diagnosis was provided, 339 were diagnosed with haematological malignancies including acute lymphoblastic leukaemia (ALL)/acute myeloid leukaemia (AML)/lymphoma, 665 patients with central nervous system (CNS) tumours including high-grade glioma (HGG), medulloblastoma and ependymoma. There were 2238 patients diagnosed with extracranial solid tumours, and 57 patients with rare cancers.

### Keywords and terminologies

In 17 studies, 9 studies incorporated a “keywords” section as part of their paper. Keywords and terminology employed in the included studies differed, which contributed to the heterogeneity of results.

A summary of the equivalent terminology derived from the included studies is presented in Supplementary Table 2. For example, terms of precision medicine were used interchangeably with “personalised medicine”, “targeted therapy”, and “molecular targeted therapy”; while terms to indicate molecular analysis included “next-generation sequencing”, “whole exome sequencing” among others.

#### Characteristics of precision medicine programmes in included studies

##### Treatment recommendations and tiering of supporting evidence

Ten studies [7, 16, 18, 19, 21–23, 25–27] described the use of a tiering system to rank the supporting evidence for PGT from the highest to the lowest, such as described earlier for the Boston tiering system (Tier 1, clinical evidence in the same cancer, all the way through to least evidence level, Tier 5, consensus opinion) [6]. This hierarchy of evidence underscores the varying degrees of confidence in treatment recommendations based on current scientific literature, however multiple systems with different standards can further complicate decision-making processes.

The 17 studies included five different tiering systems, each with between five to seven tiers, ranking the available evidence for a PGT from highest to lowest. Tiering systems included the Boston tiering system (iCAT) (4 studies; 5 tiers) [7, 19, 21, 27], OncoKB Clinical Actionability Tiers (6 tiers) [16, 26], Oxford Centre for Evidence Based Medicine guidelines [22], Level of evidence of eligible Gene Variants (6 tiers) [23], and Clinical priority score (7 tiers) [18, 25].

##### Molecular tumour board (MTB) recommendations and turnaround times

In thirteen (from 17) studies, the PGT recommendation was provided by regular MTB meetings as part of the study design, which discussed the best possible treatment plan with the molecular testing results.

Nine of the included studies reported on TAT [7, 17–21, 23–25]. The average turnaround time was 42.3 days (Table 2).

##### Molecular analyses performed

The types of molecular analysis performed varied among the included studies, as shown in Table 3. Among them, 8 studies conducted somatic whole exome sequencing (WES) [17–22, 25, 28]; 2 studies used low-coverage somatic whole genome sequencing (lcWGS) [18, 25]; somatic DNA panel sequencing was commonly used (n = 6 studies) [7, 16, 19, 22, 24, 27], among them 2 studies conducted targeted next generation sequencing (OncoPanel) of tumour DNA [7, 27]; and 9 studies performed RNA sequencing [7, 17–21, 23, 25, 26]. Four studies conducted DNA methylation profiling [18, 21, 23, 25]. Four studies performed germline whole exome sequencing (WES) [16, 19, 21, 28], and 2 studies conducted germline whole genome sequencing (WGS) [15, 26]. In one hypothetical study, a genomically guided approach was considered for treatment, as referenced in ‘You are considering recommending genomically-guided treatment’; however, no specific testing details were reported [14].

**Table 3:** Types of molecular analysis performed.

	lcWGS (Somatic)	WES (Somatic)	DNA panel sequencing	RNA-seq	DNA methylation profiling	Germline WGS	Germline WES	Others
Church et al. [7]			Targeted DNA panel (OncoPanel)	mRNA-Seq; RNA fusion panel				
Parsons et al. [23]				mRNA-Seq	DNA methylation profiling			Other: IHC assays
van Tilburg et al. [25]	lcWGS (Somatic)	WES (Somatic)		mRNA-Seq	DNA methylation profiling			RNA based gene expression array
Villani et al. [26]				mRNA-Seq		Germline WGS		Other: pan-cancer DNA panel
Ortiz et al. [22]		WES (Somatic)	Targeted gene panel					
Pincez et al. [24]			NGS DNA gene panel					Other: CGH
Mody et al. [20]		WES (Somatic)		mRNA-Seq				
Marks et al. [19]		WES (Somatic)	DNA sequencing of 467 cancer associated genes	mRNA-Seq			Germline WES	
Oberg et al. [21]		WES (Somatic)		mRNA-Seq	DNA methylation profiling		Germline WES	
George et al. [16]			circulating tumour DNA (ctDNA), Targeted NGS genetic panel				Germline WES	
Harris et al. [27]			Targeted NGS (OncoPanel)					
De Abreu Lourenco et al. [14]								Genomically guided approach, no specific information given
McCullough et al. [28]		WES (Somatic)					Germline WES	
Harttrampf et al. [17]		WES (Somatic)		mRNA-Seq				
Eaton et al. [15]						Germline WGS		Functional ex vivo drug sensitivity testing
McCarthy [29]								Genomically guided approach, no specific information given
Langenberg et al. [18]	lcWGS (Somatic)	WES (Somatic)		mRNA-Seq	DNA methylation profiling			Affymetrix gene expression
Light grey = somatic								
Dark Grey = germline								

This table depicts the sequencing test conducted by the included studies. lcWGS (Somatic), Low Coverage Whole Genome Sequencing. OncoPanel is a DNA hybrid capture-based NGS assay that detects single nucleotide variants (SNVs), insertions/deletions (indels) and copy number variants (CNVs) in cancer genes (V2: 300; V3: 447) and rearrangements in cancer genes (V2: 35; V3: 60). The OncoPanel cancer gene list was determined with input from many clinicians including paediatric oncologists and pathologists.

*WES* whole exome sequencing, *NGS* next generation sequencing, *mRNA-Seq* mRNA-sequencing, *Germline WGS* germline whole genome sequencing, *Germline WES* germline whole exome sequencing, *IHC* immunohistochemistry, *CGH* comparative genomic hybridisation.

##### Pre-clinical drug testing

Only one study reported on functional ex vivo drug sensitivity testing, with preclinical/drug testing data discussed at the MTB when treatment recommendations were formulated [15]. None of the included studies reported on patient-derived xenograft models (PDX)-related data.

##### Diagnostics and PGT clinical uptake

**Fig. 2:**
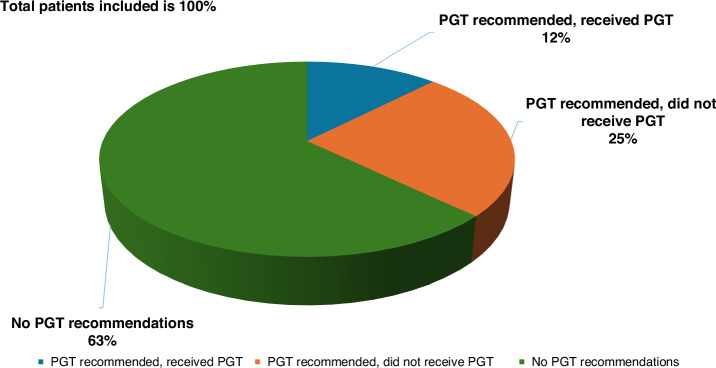
Breakdown of PGT utilisation in paediatric oncology. A total number of 3865 patients from the systematic review were included. Please see the reasons for not utilising the PGT recommendation in Fig. 3.

Thirteen studies reported on the molecular results generated for 1209 patients [7, 15, 17, 19–21, 23–27] and 311 tumour samples [16, 18] with actionable alterations. Overall, 1438 patients received formal treatment recommendations which means at least 36.2% (1438/3976) of patients had PGT options. Among the 1438 patients from the 13 included studies who had PGT recommendations, 476 patients received recommended therapy, thus the uptake rate was 33.1% (476/1438). The reported PGT adoption rate of the 13 studies that provided data ranged from 7 to 46% (median 16%). The utilisation of PGT across the included studies is illustrated in Fig. 2.

There were 1.96% patients (78/3976) whose diagnosis was changed/refined due to the molecular analysis. There were 7 studies that reported germline testing results of 10.54% (195/1849) patients with germline alterations. There was no data reported regarding the use of germline results to influence treatment recommendations and/or surveillance strategies.

##### Factors that influenced clinical uptake

**Fig. 3:**
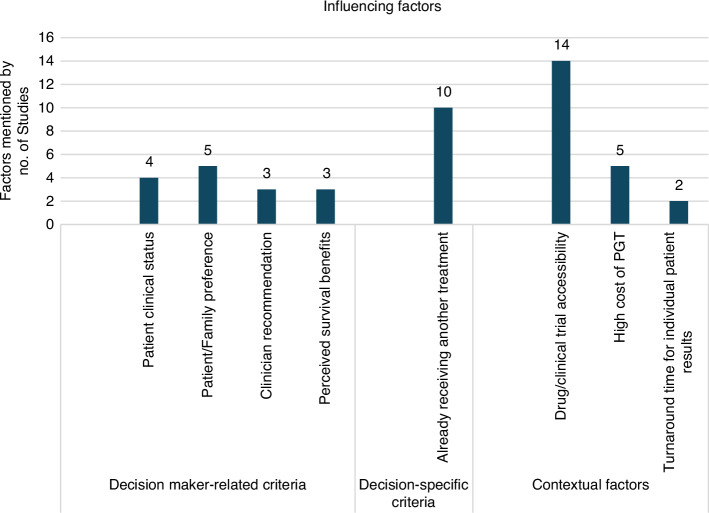
The most mentioned influencing factors. This figure depicts the most mentioned influencing factors regarding the clinical uptake of PGT within the included studies, all influencing factors were grouped by three domains.

Figure 3 presents the key factors influencing the clinical uptake of PGT, as identified across the included studies. Various factors were considered, according to the study design of included studies. For example, parents’ and oncologist perspectives of impact of genomic information on patient care were analysed [24] and themes were subsequently grouped into three domains for this systematic review: decision maker-related characteristics, decision-specific criteria, and contextual factors [30].

No studies looked at which specific factor correlated with PGT uptake. Only one DCE paper ranked the factors among different populations: healthcare professionals, patient/parents, and the general community [14].

#### Decision maker-related criteria

##### Patient clinical status

Four included studies described how patient clinical status influences the decision to take up PGT. In three studies, PGTs were not taken up to due to the patient being disease-free [20, 23, 27], stable disease [23], or well controlled by other therapy [27]. On the other hand, 15% (*n* = 31) of patients had sufficiently poor clinical status which meant PGT was not acted upon [23]. Five patients from one study who were in poor health (8.3%) died soon after sequencing was performed [24]. Three studies explored specific information regarding patient clinical status [8, 14, 20]: from 299 patients, 71 patients (23.75%) had poor performance status due to progression of underlying malignant disease, and 13 patients (4.35%) had poor performance status due to toxicity from previous treatment(s) [25]. Two studies reported clinical deterioration of the patient [16], advanced disease or death (3 patients, 16%) [27] as reasons not to pursue PGT.

##### Patient/Family preference

Five studies reported that patient/family preference affected the likelihood of PGT adoption in two directions [20, 24–26, 29]. One study described that the family/patient’s support or preference for the PGT recommendation could increase the likelihood of uptake [29]. Four studies reported that patient and/or parent preferences were important in the decision not to take up the PGT recommendation: in 2 studies, 25 patient/guardians [25], and 1 patient (1/25, 4%) did not pursue PGT due to patient/family refusal [26]; and 1 patient (1/21, 4.76%) refused PGT because of family wishes [24]. One study mentioned both family and physician preference as one of the reasons for patients not taking up (or acting on) PGT recommendations [20].

##### Clinician recommendation

Three studies offered additional insights into influencing factors for PGT uptake, related to clinician recommendation. Five patients (5/21, 23.8%) were given conventional therapy with chemotherapy or a surgical intervention as determined by their physicians [24]. Some patients did not accept PGT simply because their treating physicians thought no additional therapy was necessary [20]. Similarly, the only DCE study reported that whether the health care professional recommended the treatment to the patients affected the PGT adoption [29].

##### Patient - doctor communication barriers

One qualitative study investigated perspectives from treating physicians and parents [28]. It addressed treating physicians’ concerns that communicating complex findings from WES with parents might be disruptive during the decision-making process. This was based on the assumption that complex findings might lead to misinterpretation of the relevance of some findings, which could distract parents from making decisions in terms of cancer treatment and care for their child [28]. Despite this assumption, this study demonstrated that parents of paediatric patients with solid tumours who enroled in clinical trials have a strong desire to learn and do everything they could to support their child. Maximal provision of information was helpful for parents to make an informed decision and to take advantage of this novel technology [28].

##### Altered patient quality of life (QoL) under treatment

Two studies reported patient’s quality of life (QoL) or possible QoL after implementing the PGT recommendation as an influencing factor of uptake [14, 29].

##### Perceived survival benefits

Three studies reported that clinician perceived survival benefits [14], the severity of the patient condition without a genomic approach [29], or magnitude of the possible clinical benefits positively increase the likelihood of PGT adoption [15]. Additionally, McCarthy *et al* reported that the greater the perceived benefit to the patient, the more likely the clinician is to recommend PGT [29].

#### Decision-specific criteria

##### Already receiving another treatment

Four studies reported receiving standard treatment as one of the reasons that patients did not implement PGT recommendation. These four studies referred to standard treatment, conventional treatment, first-line therapy, proven standard relapse therapies, and frontline treatment, which were distinguished from clinical trials [7, 16, 24, 25]. In one study, 200 patients (48%) were not expected to consider PGT as they were newly diagnosed, indicating that standard treatment was the preferred option. Despite experiencing relapse/refractory disease, only 4 (7%) out of 57 patients took up PGT recommendations, with a considerable proportion of patients (no specific number given here) receiving conventional therapy after they underwent sequencing [16].

Six studies reported patients were already receiving other alternative treatment [3, 9–11, 18, 22] as a reason to not take up PGTs. One quantitative study reported that the most common reason for not adopting PGT was receipt of other treatment in 32% (64/200) patients in their study [23]. Two studies mentioned that PGTs were saved for a later time point as alternate treatment [16, 17].

Other alternative treatment here means therapies including no cancer-directed systemic therapy for 49/204 (24%) patients, enrolment on a phase I/II clinical trial which did not require biomarker sequencing [16], attempting third-line therapy (other non-PGT experimental therapy) [27], or other alternative therapies with no further information [15, 17, 23]. One quantitative study reported that an alternative regimen was chosen for 14.67% (11/75) of patients [17].

##### Turnaround time for individual patient results

Two studies reported that patients did not have a chance to implement PGT due to the turnaround time for sequencing results and a treatment recommendation. Some patients died before finalisation of the molecular analysis and molecular tumour board report (*n* = 22, 7.36%) [25] or were in palliative care due to the testing results being generated too late (mean TAT = 54 days, range, 15-114 days) in the clinical course [20].

##### Tier level of supporting evidence

Ten (out of 17) studies included tiering systems regarding level of supporting evidence for treatment recommendations [7, 16, 18, 19, 21–23, 25–27]. Only one study reported that patients who received the highest level of recommendation, referred to as a Tier 1 recommendation in the iCat recommendation tiering system, were more likely to adopt PGT when compared to patients who did not receive a top-tier recommendation [7]. The rest of the 9 studies did not provide this information related to treatment decision-making.

#### Contextual factors

##### Drug/clinical trial accessibility

Fourteen out of 17 included studies (82.4%) reported that the accessibility of treatment and/or drugs was a significant issue influencing uptake of PGTs.

Five studies described patient-related factors and/or clinical trial eligibility that can affect access to drug treatment. In some cases, patients were not eligible to adopt the PGT due to clinical trials restrictions [22], persistent metabolic disorders [24] or inappropriate current clinical status [27]. Harttrampf et al. [17]. reported 13 patients (30.95%) had no access to a clinical trial because of age, timing of trial opening, no respective age slot available, or trial inclusion criteria not fulfilled (i.e. patient not eligible for the trial).

Six studies reported that PGT uptake was hindered by lack of an available drug or clinical trial [7, 16, 20, 24–26]. Two studies highlighted that numerous new drugs have not yet been approved for use in children due to insufficient data on their effectiveness in treating paediatric diseases or their safety in children [17, 21]. Furthermore, the process of obtaining experimental drugs that are not approved by the FDA (or relevant national drug approval system) for paediatric patients is time-consuming, and access to these drugs is rarely granted to younger patients [19, 21]. Experimental medicines being tested in clinical trials or newly licensed drugs for adults are rarely given to young patients, even in cases when compassionate use is considered [21]. For 11 (out of 299, 3.68%) patients, the drug formulation was unavailable despite the targets being identified [25].

##### High cost of recommended treatments

Two studies described cost as a major barrier to the implementation of PGT, despite the reduced cost of sequencing [25], as the personnel cost of precision medicine is expected to remain high [19].

Once a PGT recommendation is made, the cost of drug was also a barrier to access, as described in five studies [14, 16, 21, 25, 29]. Five studies reported cost-related factors that ranged from (total) cost of treatment [14, 29], insurance company declined cost coverage [25], and the off-label use of high-cost agents not covered by insurance companies [14, 21, 29]. These factors can all directly impact the adoption of PGT [14, 29]. Patients did not adopt PGT due to difficulties accessing novel drugs on a compassionate-use basis, which is cost-related, with no specific patient numbers provided [16].

## Discussion

To the best of our knowledge, this is the first systematic review to assess the existing evidence for factors that influence clinical uptake of PGT for paediatric oncology patients. This review has identified and grouped the key influencing factors into three domains: decision maker-related characteristics, decision-specific criteria, and contextual factors [30]. Our findings align with and extend the framework of decision-making factors in oncology: receiving other treatment, turnaround time, tiering of recommendations, patient and doctors’ perspectives. Ethical and communication challenges also emerged, particularly when conveying complex or uncertain results to families. Notably, a gap exists between clinicians’ concerns about overwhelming families and parents’ desire for detailed information and involvement in decisions. These findings highlight the need for better communication strategies and support systems to facilitate informed decision-making in PGT. Understanding these influencing factors is essential for optimising the implementation of PGT and in determining the relative importance of components of precision medicine such as pre-clinical testing in decision-making. Understanding how these factors intertwine to impact the individual decision-making unit for each child (clinician paired with patient and family) will be important to facilitate the most supportive system of care for children with cancer. Across the 17 included studies, a consistently low uptake rate of PGT was observed, ranging from 7% to 46%.

While PGT may offer potential benefits, it is often considered a ‘last option’ for patients in practice. Reasons include the limited availability of clinical data in paediatric cancer, challenges in combining targeted agents with traditional chemotherapy, and issues with insurance approval for targeted therapies. For instance, high-risk patients who are in remission from first-line therapy, radiation, or surgery, or relapsed patients already undergoing second-line chemotherapy, may not be candidates for PGT even when genomic analysis suggests its potential benefit. One recent study highlighted that one of the key challenges was convincing oncologists of the value of precision oncology in patient care. This challenge could be partly overcome through more evidence in paediatrics [5], educational opportunities and integrating precision oncology into weekly Tumour Board discussions [31].

Among decision-specific criteria, three factors we identified could be addressed to facilitate the uptake of PGT in paediatric cancer: receiving other treatment, terminology and tiering of evidence for a treatment recommendation.

This review adds to the literature by identifying interlinked factors from decision maker-related criteria and decision-specific criteria. These interlinked factors could decrease the likelihood of uptake of PGT. Using an example with “turnaround time”, “patient clinical status” and “receiving other treatment”, the longer turnaround time might lead to worse patient clinical status and more patients receiving another treatment. For patients with aggressive underlying cancer, they may miss the chance to take up PGT before further disease progression.

Our review also identified interlinked influencing factors including patient clinical status, which has potential to impact clinical uptake in two directions. Further investigations/research are needed to explore detailed impact of each factor and its relative importance.

Across our analysis, a predominant factor for not adopting or delaying PGT was the pursuit of other treatments, indicating that PGT is usually adopted late following conventional treatments. Furthermore, the toxicity of prior treatments may affect adoption of PGT. In some cases, PGT may be preferred by patients fortunate enough to navigate through conventional treatments with minimal side-effects. The exact timing of PGT uptake, if taken up at all, will be impacted by factors including patient clinical status, drug/clinical trial accessibility, perceived survival benefits, and altered patient QoL under treatment. It is important to note that current paediatric oncology studies using precision medicine mainly focus on high-risk, rare cancers, where standard treatments are often insufficient. For these patients, PGT may offer a more viable treatment option. A recent publication indicated that patients who received PGT earlier in the disease course had greater survival benefit [5].

Specific examples of how PGT could be further facilitated where appropriate may include starting molecularly targeted therapies upfront for patients with a significantly high risk of relapse with standard therapy [31].

Regarding decision maker-related characteristics, we found both clinician support and patient or parent preference affect the uptake of a PGT recommendation. However, only five studies mentioned patient or parent preference as a factor, as this was not recorded in the clinical records. This reflects the need for qualitative research to investigate stakeholders’ perspectives in uptake of PGT recommendations. For instance, interviews and thematic analysis studies would be helpful in exploring patient or parent decision-making experience.

Although PGT shows promise for paediatric cancer patients, its uptake often occurs after all available conventional therapies have been tried. This approach may lead to increased toxicity with each successive treatment [4, 31]. Since patient clinical status is another significant factor that affects the PGT adoption, and one of the reasons to discontinue PGT, early adoption of PGT could be encouraged at an organisational or policy level for patients who are less likely to benefit from conventional therapy [32].

Understanding treatment toxicity is essential when adopting new interventions, yet current literature lacks consistent reporting on toxicity profiles and their impact on perceptions of PGT. Comprehensive toxicity data and reporting of actionable pharmacogenomic variants, such as through a national Australian randomised-control trial MARVEL-PIC [33], may help integrate expected toxicities into clinical decision-making and support timely adoption of PGT. Outside of clinical trials, real-world studies such as SACHA-France study (NCT04477681), which collects safety and efficacy data for off-label or compassionate use therapies in patients under 25 years, could help build an evidence base for toxicity profiling for PGT in children [34].

Current barriers, such as turnaround time and cost-related factors are expected to decrease with the rapid development of medical and genomic technology. Faster turnaround time may provide more chance for patients to try PGT while they are clinically well enough or before going on to palliative care.

Among contextual factors, costs remain a significant barrier to the implementation of PGT in some countries. Furthermore, it is important to highlight that most of the included studies were clinical trials, which provided patients with access to medications. As a result, the reported uptake rates may not accurately represent the barriers encountered in real-world settings.

This systematic review has several important implications. The complexity of cancer treatment/care is increasing due to the rapid development of biomedicine, novel techniques, genetic research, drug discovery and advancements in cancer therapies [35, 36]. To enhance patient and family understanding of these novel techniques and treatments, a patient-friendly glossary of precision medicine terms and supportive visual resources are essential [37]. More qualitative research is needed to investigate the perspectives of primary decision-makers: treating oncologists and patients/parents/family members. Exploring clinicians’ experience regarding precision medicine, MTB meetings and making recommendations for PGT could greatly extend our current knowledge of the treatment decision making process. By understanding these perspectives, potential communication barriers can be identified and addressed, leading to better-informed decision-making, improved patient outcomes, and enhanced efforts to reduce barriers. A DCE survey approach with clinical and parent decision makers would be useful to explore the relative importance of each influencing factor. Our review found that most studies (13 out of 17) employed MTBs, although the impact of MTBs on uptake of PGT is unknown. We hypothesise that MTBs could facilitate the implementation of precision medicine in paediatric cancer and broader diseases. By leveraging the expertise of a multidisciplinary team, employing MTBs can enhance the understanding and implementation of PGT, which is aligned with recent precision medicine MTB studies [38, 39]. This collaborative approach can ensure that various perspectives and specialised knowledge are integrated into treatment planning, thereby improving patient outcomes.

One barrier to the implementation of genomic testing is the confidence of oncologists in ordering, interpreting, and utilising the results in treatment decisions, as documented in adult oncology [40, 41]. Although the application of precision oncology in paediatric cancer has progressed more slowly than in adults, similar findings have emerged [42]. While this is a documented barrier, none of our included studies have reported this factor. Future clinical studies should focus on identifying and addressing clinician confidence to improve the integration of precision medicine in paediatric cancer care.

Given the complexity and sheer volume of data generated through genomic sequencing—and the limited confidence many oncologists have in interpreting genomic information, expert review is essential to help translate PGT results into clinical decisions [43]. To address these factors, we believe that MTBs play a pivotal role in precision oncology by synthesising genomic data and providing precision-guided treatment (PGT) recommendations to clinicians [44]. However, not all included studies used MTBs in their study, and no study has systematically examined how paediatric oncologists make treatment decisions after receiving MTB recommendations. Future studies should address this gap and explore the resources, support, and training needs of oncologists in the context of precision medicine.

The limited scope of available research in this area underscores the need for future research to validate and expand upon the current findings. One limitation of this review was the paucity of articles that provided detailed quantitative analysis of key factors which affected PGT uptake in precision oncology. The focus of most included studies was not decision-making, so our data was taken from studies where this was a secondary aim. While the uptake of PGT recommendations may differ between newly diagnosed and relapsed paediatric cancer patients [45], our a priori concept was to include both populations. After conducting our systematic review search, it became clear that there is limited literature to be able to differentiate these groups further. Some studies note that newly diagnosed patients are less likely to implement PGT or enrol in PGT-directed clinical trials [7]. Both newly diagnosed and relapsed patient populations were included to provide a broader understanding of current trends, with this inclusion acknowledged as a limitation. Future studies could explore these differences as more data becomes available.

Additionally, there are reasons not specified as most influencing factors reported were part of the patients’ clinical report/record, which means there are cases where PGT was not adopted without specific reasons provided [25]. Therefore, there is a need for both quantitative and qualitative studies in this field, as quantitative research could compare the clinical benefits between PGT recommendation and conventional therapy, and the qualitative studies could explore more detailed influencing factors on treatment decision making in paediatric precision oncology.

Meaningful comparisons across studies remain challenging due to heterogeneity in study design, variations in terminology, differences in tiering of evidence, and inconsistent use of MTBs. Among the 17 studies reviewed, diverse terminology and tiering systems were used, and the role and structure of MTBs varied considerably. This variability limits the synthesis of findings and the generalisability of evidence in the precision medicine literature. Standardisation of evidence classification and tiering systems may support treating oncologists in interpreting and implementing research findings more effectively [9, 32, 46].

While our findings do not directly demonstrate that the lack of harmonisation in tiering systems impacts the implementation of PGT recommendations, this variability presents challenges for comparing MTB recommendations across institutions and regions. Notably, an Australian study found that Tier 1 (higher-tier) recommendations were associated with greater uptake of PGT, suggesting that clearer classification may support implementation [5]. Harmonising tiering systems could therefore enhance the clarity and clinical utility of MTB reports; and improve the consistency and comparability of future studies. The absence of a standardised approach also limits cross-study evaluation and may affect clinician confidence in interpreting and applying MTB recommendations. Coordinated efforts toward a unified tiering framework would support clearer communication and facilitate broader comparative analysis.

As evidence for precision-guided treatment (PGT) continues to evolve—particularly in paediatric oncology—it is critical to establish consistent frameworks for evaluating efficacy, side effects, and other clinical outcomes. This will help make the clinical utility of PGT more accessible to healthcare professionals and promote its integration into routine care [47]. Furthermore, the adoption of standardised terminology and tiering systems could facilitate international collaboration and accelerate global research efforts. Inconsistent medical terminology can impact professional communication and ability to conduct cross-study comparisons, which may make doctor–patient discussions more challenging, in turn potentially limiting understanding and uptake of emerging technologies [37] Addressing these challenges is essential to support equitable and effective implementation of PGT across diverse clinical settings.

## Conclusion

This systematic review has synthesised available data to identify key factors influencing paediatric cancer treatment decision-making and the adoption of PGT. The current literature highlights influencing factors across three domains: decision maker-related characteristics, decision-specific criteria, and contextual factors. Among key influencing factors, interlinked factors, already receiving another treatment, turnaround time, and patient clinical status were identified across decision maker-related criteria and decision-specific criteria. The preferences of both clinicians and patients/parents can impact the adoption of PGT. However, more detailed influencing factors remain to be explored. This knowledge has the potential to shape and facilitate the implementation of precision medicine in cancer care and more broadly outside of cancer. Further research is necessary to elucidate the hierarchy of decision-making factors in PGT and to determine what is most valued by clinicians, patients, and parents.

## Supplementary information

Supplementary tables
