# Supplementary material for: Factors that influence the uptake of precision-guided treatment recommendations in paediatric cancer: a systematic review
Source: Br J Cancer. 2025 Sep 24;134(1):8–21. doi: 10.1038/s41416-025-03171-6 (PMC12764839; doi:10.1038/s41416-025-03171-6)
Supplement: Supplementary file 1 — Supplementary tables [file 41416_2025_3171_MOESM1_ESM.docx]

**SUPPLEMENTARY**

| **Supplementary Table 1: Quality assessment of included studies using Mixed Methods Appraisal Tool (MMAT)** | | | | | | | | | | | | | | | | | | | | | | | | | |
| --- | --- | --- | --- | --- | --- | --- | --- | --- | --- | --- | --- | --- | --- | --- | --- | --- | --- | --- | --- | --- | --- | --- | --- | --- | --- |
| **First author, Year** |  |  | 1. Qualitative | |  | 2. Quantitative randomized controlled trials | | | | |  | 3. Quantitative nonrandomized | | | |  | 4. Quantitative descriptive | | |  |  | 5. Mixed methods | | |  |
|  | 1.1 | 1.2 | 1.3 | 1.4 | 1.5 | 2.1 | 2.2 | 2.3 | 2.4 | 2.5 | 3.1 | 3.2 | 3.3 | 3.4 | 3.5 | 4.1 | 4.2 | 4.3 | 4.4 | 4.5 | 5.1 | 5.2 | 5.3 | 5.4 | 5.5 |
| De Abreu Lourenco, 2021 | |  |  |  |  |  |  |  |  |  | Y | Y | Y | Y | Y | |  |  |  |  |  |  |  |  |  |
| Eaton, 2022 |  |  |  |  |  |  |  |  |  |  |  |  |  |  |  | Y | Y | Y | Y | Y |  |  |  |  |  |
| George, 2019 |  |  |  |  |  |  |  |  |  |  | Y | Y | Y | Y | Y | |  |  |  |  |  |  |  |  |  |
| Harris, 2016 |  |  |  |  |  |  |  |  |  |  |  |  |  |  |  | Y | Y | Y | Y | Y |  |  |  |  |  |
| Harttrampf, 2017 |  |  |  |  |  |  |  |  |  |  |  |  |  |  |  | Y | Y | Y | Y | Y |  |  |  |  |  |
| Langenberg, 2022 |  |  |  |  |  |  |  |  |  |  |  |  |  |  |  | Y | Y | Y | Y | Y |  |  |  |  |  |
| Marks, 2017 |  |  |  |  |  |  |  |  |  |  |  |  |  |  |  | Y | Y | Y | Y | Y |  |  |  |  |  |
| McCarthy, 2020 | Y | Y | Y | Y | Y |  |  |  |  |  |  |  |  |  |  |  |  |  |  |  |  |  |  |  |  |
| McCullough, 2016 | Y | Y | Y | Y | Y |  |  |  |  |  |  |  |  |  |  |  |  |  |  |  |  |  |  |  |  |
| Mody, 2015 |  |  |  |  |  |  |  |  |  |  |  |  |  |  |  | Y | Y | Y | Y | Y |  |  |  |  |  |
| Oberg, 2016 |  |  |  |  |  |  |  |  |  |  |  |  |  |  |  | Y | Y | Y | Y | Y |  |  |  |  |  |
| Ortiz, 2016 |  |  |  |  |  |  |  |  |  |  |  |  |  |  |  | Y | Y | Y | Y | Y |  |  |  |  |  |
| Parsons, 2022 |  |  |  |  |  |  |  |  |  |  |  |  |  |  |  | Y | Y | Y | Y | Y |  |  |  |  |  |
| Pincez, 2017 |  |  |  |  |  |  |  |  |  |  |  |  |  |  |  | Y | Y | Y | Y | Y |  |  |  |  |  |
| vanTilburg, 2021 |  |  |  |  |  |  |  |  |  |  | Y | Y | Y | Y | Y | |  |  |  |  |  |  |  |  |  |
| Villani, 2022 |  |  |  |  |  |  |  |  |  |  |  |  |  |  |  | Y | Y | Y | Y | Y |  |  |  |  |  |
| Church, 2022 |  |  |  |  |  |  |  |  |  |  | Y | Y | Y | Y | Y | |  |  |  |  |  |  |  |  |  |

**Supplementary Table 2**. Keywords listed from the included studies (terminology used)

|  | Keywords using | | | |
| --- | --- | --- | --- | --- |
|  | **Precision medicine** | **Molecular analysis** | **Clinical decision making** | **Cancer/childhood cancer** |
| Ortiz et al. 2016 |  | Molecular biology |  | Paediatric oncology; |
| Pincez et al. 2017 |  | Next-generation sequencing; Molecular profiling; Array comparative genomic hydridization |  | Solid tumor |
| Marks et al. 2017 | Targeted therapy | Next-generation sequencing; Genomic |  | Pediatric leukemia; Hematologic malignancies, |
| Oberg et al. 2016 | Precision medicine | RNA sequencing; Whole exome sequencing |  | Paediatric oncology |
| George et al. 2019 | Personalised medicine | Clinical targeted sequencing; Circulating tumour DNA |  | Paediatric oncology |
| De Abreu Lourenco et al. 2021 |  | Next-generation sequencing; Genomics | Choice; Decision-making; Preferences | Childhood cancer |
| McCullough et al. 2016 |  | Whole exome sequencing |  | Childhood cancer |
| Eaton et al. 2022 | Precision medicine | Genomic profiling; Drug screening assays; |  | Paediatric oncology; Acute lymphoblastic leukemia; Acute myeloid leukemia; Antitumor, |
| Langenberg et al. 2022 | Precision medicine; Molecular targeted therapy | Molecular biology; Next-generation sequencing |  | Cancer; Child; Adolescent |
